# Trait anxiety and valence-arousal preferences in music among Chinese college students

**DOI:** 10.3389/fpsyg.2026.1859454

**Published:** 2026-10-20

**Authors:** Ya Ting Huang, Fu Qiang Yuan

**Affiliations:** Jiangxi University of Chinese Medicine, Nanchang, China

**Keywords:** arousal, college students, individual differences, music preference, trait anxiety, valence

## Abstract

Music preference may shape how individuals engage with emotionally characterized music, yet the association between trait anxiety and preference for music with different affective properties remains unclear. This study examined preference ratings for 90 lyric-free music excerpts drawn from a validated stimulus database in a formal sample of 90 Chinese college students. Participants were selected from a larger screening pool and were classified into high-, medium-, and low-trait-anxiety groups for the primary analysis. Each participant rated 30 positive-, 30 neutral-, and 30 negative-valence excerpts. Importantly, the three stimulus categories differed in both valence and arousal; therefore, the categories are interpreted here as valence-defined, arousal-differentiated stimulus sets rather than as a pure manipulation of valence. Repeated-measures ANOVA showed a significant stimulus-category effect and a significant trait-anxiety-group x stimulus-category interaction. The low-anxiety group gave relatively higher ratings to the positive category, whereas the medium-anxiety group rated the negative category higher than the low-anxiety group; no between-group difference was observed for the neutral category. Males showed higher overall preference ratings than females, while the gender x stimulus-category interaction was not significant. The study measured trait anxiety and music preference only; it did not directly assess mood change, emotion-regulation goals, therapeutic effects, or neurobiological mechanisms. Accordingly, the findings are interpreted as group-based associations within the tested stimulus set, with arousal, participant categorization, and stimulus-level variability identified as important limitations.

## 1 Introduction

Trait anxiety is a relatively stable individual difference in the tendency to perceive situations as threatening and to respond with elevated anxiety. Music preference is also an individual-difference construct, reflecting systematic variation in what listeners choose or rate favorably across musical contexts (Rentfrow and Gosling, 2003; Chamorro-Premuzic and Furnham, 2007). Music listening is commonly used in everyday coping, but the function of music depends partly on the listener and on the properties of the music itself (Ferreri et al., 2021). Accordingly, the present study distinguishes the directly measured outcome of music-preference ratings from broader, unmeasured constructs such as emotional regulation and symptom change.

A central methodological issue is the distinction between musical emotion, emotional valence, arousal, and preference. Juslin and Västfjäll (2008) emphasized that emotional responses to music can arise through multiple mechanisms and should not be equated with liking. Russell's (1980) circumplex model likewise treats valence and arousal as separable dimensions of affective experience. Classic accounts of activation further indicate that arousal can influence evaluation and behavior (Hebb, 1955; Schachter and Singer, 1962). In music research, Ho and Loo (2023) further proposed that arousal and valence interact with tempo, familiarity, preference, and the presence of lyrics, underscoring the need to interpret these attributes jointly. Broader performance-oriented accounts also emphasize the regulation of activation or energy states (Loehr and Schwartz, 2003), although such accounts are not direct models of music preference. These perspectives make it necessary to avoid interpreting a category difference as a valence effect when arousal also differs between categories.

Theoretical accounts such as self-verification and mood management may help generate hypotheses about why people sometimes prefer affect-congruent or affect-incongruent material (Swann et al., 1992; Zillmann, 1988). Research on sad music similarly shows that preference for negatively valenced music cannot be reduced to a wish to intensify negative mood; low energy, familiarity, aesthetic appraisal, and individual characteristics can also contribute (Arens and Stangier, 2020; Yoon et al., 2020; Wang et al., 2018). These theories are therefore used in the present manuscript as interpretive background rather than as directly tested mediating mechanisms.

Previous music-preference research has frequently relied on genre labels or self-reported listening habits, which can obscure the affective and acoustic characteristics of the actual stimuli (Dunn et al., 2012; Vella and Mills, 2017). Experimental work using musical excerpts demonstrates that preference and stress-related responses depend on both stimulus characteristics and personal preference (Jiang et al., 2013, 2016). The present study therefore used actual lyric-free excerpts and collected ratings of valence, arousal, familiarity, and preference. Cultural origin was balanced in the formal stimulus set and familiarity was checked across categories. However, arousal was not matched across the final valence-defined categories, and quantitative tempo values were not included in the inferential analysis. This constraint is treated as a substantive limitation rather than as evidence that valence was isolated.

The study had two empirical aims: first, to construct and describe a set of Chinese traditional/folk and Western classical instrumental excerpts with subjective affective annotations; second, to examine whether preference ratings across the resulting valence-defined stimulus categories differed among college students with different levels of trait anxiety. Gender was examined as an exploratory between-subjects characteristic. The design was not an intervention and no pre-post measure of anxiety or mood was collected. Thus, the study addresses associations between trait anxiety and music preference, not whether music regulates anxiety or produces therapeutic benefit.

Based on the preceding literature and the measurement framework summarized in Figure 1, the following hypotheses were evaluated in the formal preference experiment.

**Figure 1:**
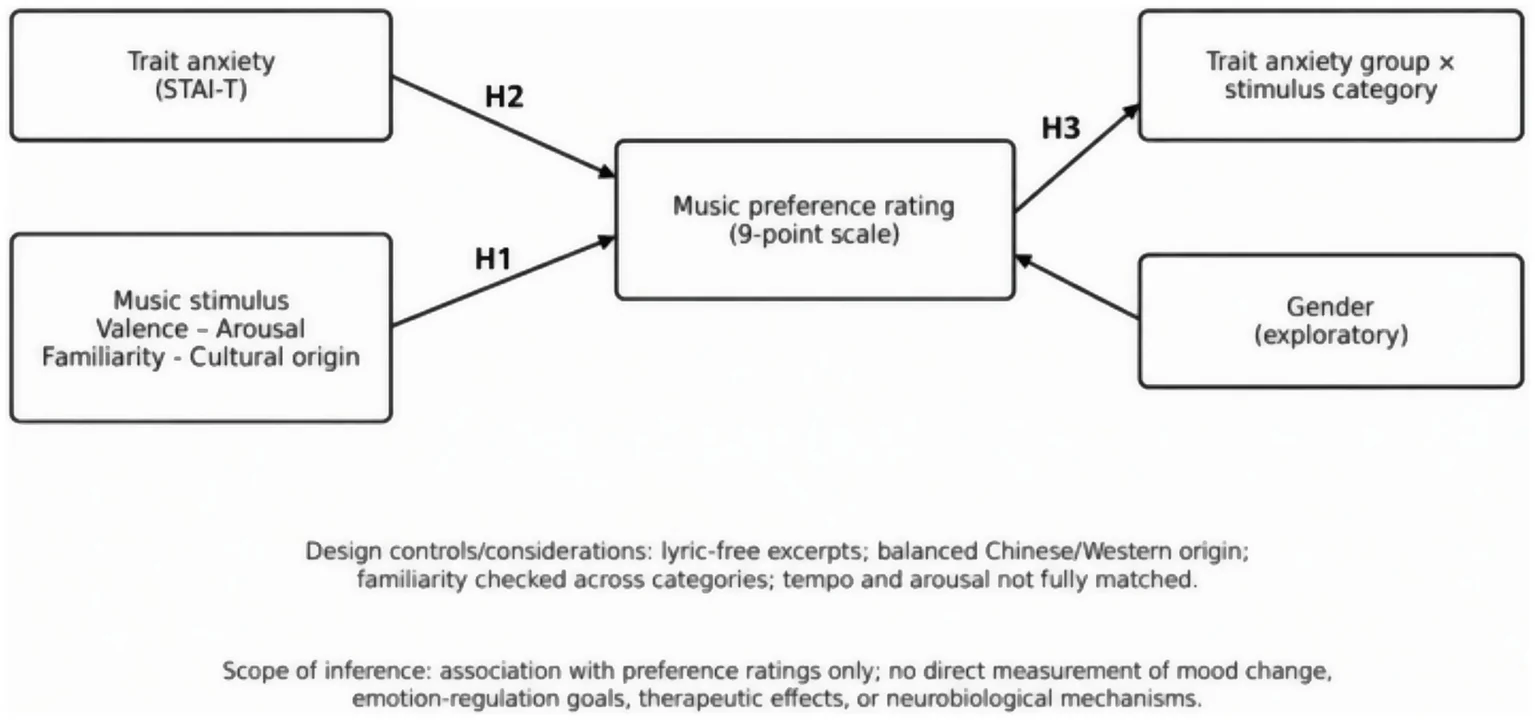
Conceptual and measurement framework for trait anxiety and music preference.

### 1.1 Hypotheses

H1. Preference ratings will differ across the three valence-defined stimulus categories, with the positive category receiving higher ratings than the negative category.

H2. Trait-anxiety group will interact with stimulus category, such that the positive-to-negative preference contrast will vary across high-, medium-, and low-trait-anxiety groups.

H3. Between-group differences will be smaller for the neutral category than for the positive and negative categories.

## 2 Method

The study comprised two linked components: construction of an emotional music stimulus database and a formal online music-preference experiment. More than 2,000 lyric-free instrumental pieces were initially considered. After content and technical screening, 633 excerpts entered subjective evaluation and 553 met the final quality criteria. A separate group of 185 college students provided affective ratings for stimulus validation. The formal preference experiment used 90 excerpts (30 per valence-defined category) and included 90 college students. The analysis reported below is based on the formal experimental sample; the larger screening pool was used only to identify eligible participants and did not complete the 90-excerpt preference task.

### 2.1 Construction and validation of the emotional music stimulus database

#### 2.1.1 Objectives and theoretical foundations

The stimulus database was organized with reference to Russell’s (1980) circumplex model of affect and the Self-Assessment Manikin (SAM; Bradley and Lang, 1994). Valence and arousal were treated as the core affective dimensions. Familiarity was collected as a separate study-specific rating and was not treated as a standard SAM dimension. This distinction is important because familiarity reflects prior exposure or recognition, whereas SAM was used to index affective valence and arousal. Theoretical accounts of music-evoked emotion and the valence-arousal relationship (Juslin and Västfjäll, 2008; Ho and Loo, 2023) informed the decision to retain both affective dimensions in the description of the stimulus set.

#### 2.1.2 Music collection and preprocessing

More than 2,000 lyric-free instrumental music pieces were initially collected from Chinese and Western musical traditions. Materials containing linguistic lyrics, highly familiar commercial melodies, distorted or incomplete audio, or unclear tonality were excluded. The remaining excerpts were organized to maintain approximate balance between Chinese traditional/folk music and Western classical music. After initial screening, 633 excerpts were edited into 15-s clips, subjected to noise reduction, and normalized for loudness. Clips with peak distortion or substantial background noise were removed. The exclusion of lyrics controlled the presence-of-lyrics factor identified as relevant to valence-arousal judgments (Ho and Loo, 2023). Tempo was considered during preliminary stimulus organization but was not retained as a quantitative covariate in the inferential dataset.

#### 2.1.3 Emotional classification and annotation

The retained excerpts were preliminarily annotated by musical and affective characteristics, including emotional label, mode or tonality, apparent tempo, and interval pattern. The affective annotation was guided by dimensional accounts of valence and arousal and by research on music-evoked emotion (Russell, 1980; Juslin and Västfjäll, 2008), whereas the descriptive organization of cultural origin, instrumental genre, musical form, and historical period followed standard musicological treatments of Chinese and Western musical traditions (Lau, 2008; Burkholder et al., 2019). These annotations were used for stimulus organization rather than as substitutes for subjective ratings. Because a musical excerpt can be pleasant but low in activation, or unpleasant but highly activating, the final formal categories were defined primarily from participant ratings and are described below in terms of both valence and arousal. Figure 2 summarizes this organizational framework and the metadata dimensions used during stimulus preparation.

**Figure 2:**
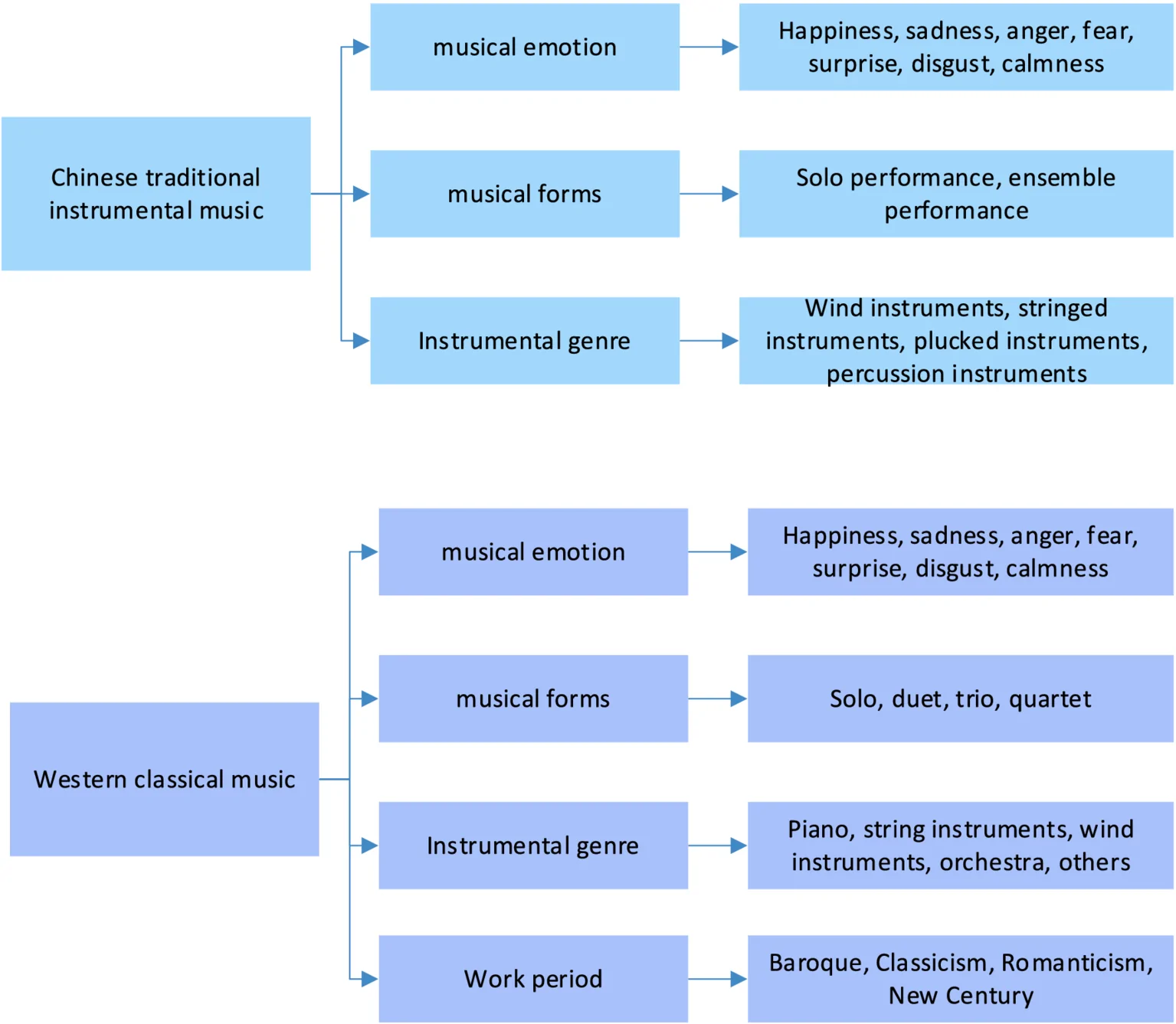
Preliminary organizational scheme for Chinese and Western instrumental music stimuli.

#### 2.1.4 Subjective rating, familiarity assessment, and stimulus classification

A total of 185 university students who did not participate in the formal preference experiment evaluated the candidate excerpts. Valence and arousal were rated using 9-point SAM-based scales. Familiarity was assessed separately with a study-specific 9-point item ranging from very unfamiliar to very familiar. Familiarity was therefore a control-related descriptive measure, not a SAM subscale. Excerpts with more than 10% missing ratings, an across-rater standard deviation greater than 2.00 for valence, or technical loudness inconsistency after normalization were excluded. These criteria reduced the candidate set from 633 to 553 validated excerpts. Because each affective dimension was represented by a single judgment rather than a multi-item questionnaire, Cronbach’s alpha was not used as an index of internal consistency for the stimulus ratings; quality control instead relied on missingness, across-rater dispersion, technical screening, and between-category separation.

(1) Positive-valence category: mean valence rating ≥ 6.50 and mean arousal rating ≥ 4.50;
(2) Neutral-valence category: 3.50 < mean valence rating < 6.50, without an extreme positive or negative valence profile;
(3) Negative-valence category: mean valence rating ≤ 3.50 and mean arousal rating ≥ 4.50.

The three categories differed significantly in valence and also differed significantly in arousal (both *p* < 0.001), whereas familiarity did not differ across the final formal categories (*p* = 0.98). Accordingly, the categories should not be interpreted as isolating valence independently of arousal. In subsequent sections, the term valence-defined stimulus category is used to preserve the original classification while making the arousal difference explicit.

#### 2.1.5 Quality control and database compilation

After technical screening and subjective-rating quality checks, the final database comprised 553 excerpts: 289 Chinese traditional/folk excerpts and 264 Western classical excerpts. Each excerpt was annotated with cultural origin and the available affective ratings. The formal 90-excerpt set was drawn from this database while balancing Chinese and Western origin within each category. Because familiarity did not differ across the three formal categories, familiarity was less likely to explain category-level preference differences. By contrast, arousal did differ across categories and therefore remained a potential contributor to the observed preference pattern.

The database should therefore be viewed as a stimulus resource with documented valence, arousal, familiarity, and cultural-origin properties rather than as a set in which one affective dimension was experimentally isolated. This interpretation is consistent with the multidimensional nature of musical affect and with the present study’s correlational preference objective.

### 2.2 Preferences across valence-defined music categories among college students with trait anxiety

#### 2.2.1 Participants

College students were recruited online nationwide through the BrainIsland platform. A total of 519 individuals completed the screening questionnaire, which included demographics, the trait-anxiety component of the State–Trait Anxiety Inventory (STAI-T; Spielberger et al., 1983; Li and Qian, 1995), the Patient Health Questionnaire-9 (PHQ-9; Kroenke et al., 2001), and a brief music-genre preference item. Participants with PHQ-9 scores greater than 10 were excluded (*n* = 78); thus, a score of exactly 10 was retained and the retained pool should not be described as entirely free of moderate depressive symptoms. A further 41 respondents were excluded because they were not eligible college students or provided incomplete responses, and 27 were excluded because they met the prespecified operational definition of a strong preference for one music genre. Strong genre preference was defined as rating one genre at least 8 on a 9-point scale while rating the next preferred genre at least 3 points lower. This exclusion was intended to reduce dominance by a single pre-existing genre preference, but it also narrows the population to which the findings generalize.

After screening, 373 participants remained eligible. These 373 participants constituted the screening pool only; they did not all complete the music-preference experiment. Kelley’s (1939) extreme-group method was used to define upper and lower trait-anxiety bands: the top 27% were classified as high trait anxiety, the bottom 27% as low trait anxiety, and the middle 46% as medium trait anxiety. Thirty participants were randomly selected from each band for the formal 90-excerpt experiment, yielding *N* = 90 (41 males, 49 females). The remaining screening participants did not receive the formal music task and therefore have no music-preference outcome data. The use of grouped STAI-T scores provides a direct comparison among predefined bands but discards information from a continuous individual-difference variable; this limitation is considered explicitly in the interpretation. Group mean ages and STAI-T scores were: high group, age *M* = 21.43 years (*SD* = 2.05), STAI-T *M* = 53.27 (*SD* = 8.62); medium group, age *M* = 22.23 (*SD* = 2.42), STAI-T *M* = 36.57 (*SD* = 1.71); low group, age *M* = 21.33 (*SD* = 1.71), STAI-T *M* = 25.87 (*SD* = 3.89). Group STAI-T scores differed significantly, *F*(2, 87) = 180.81, *p* < 0.001.

#### 2.2.2 Experimental materials

Ninety excerpts were selected from the validated database: 30 positive-valence, 30 neutral-valence, and 30 negative-valence excerpts, with 15 Chinese and 15 Western excerpts in each category. The positive category had mean valence ≥ 6.50 and mean arousal ≥ 4.50; the negative category had mean valence ≤ 3.50 and mean arousal ≥ 4.50; and the neutral category was drawn from the middle valence range. The selected categories differed in valence, *F*(2, 87) = 351.36, *p* < 0.001, and arousal, *F*(2, 87) = 289.52, *p* < 0.001. Familiarity did not differ, *F*(2, 87) = 0.12, *p* = 0.98. Equal Chinese/Western representation and the familiarity check reduced two potential confounds, but the significant arousal difference means that any category effect may reflect valence, arousal, or their combination.

Trait anxiety was assessed with the trait-anxiety component of the State–Trait Anxiety Inventory, hereafter consistently abbreviated STAI-T (Spielberger et al., 1983). The Chinese college-student adaptation was reported by Li and Qian (1995). The scale uses Likert-type responses, with higher total scores indicating greater dispositional anxiety. Prior Chinese validation work reported strong internal consistency. The inferential dataset available for the present report contained scale totals rather than the complete item-level response matrix; therefore, a sample-specific Cronbach’s alpha could not be recomputed and is not reported as though it had been estimated in this sample.

The PHQ-9 was used as a screening measure for depressive symptoms because depression can be associated with preferences for low-energy or sad music (Arens and Stangier, 2020; Yoon et al., 2020). The PHQ-9 contains nine items rated from 0 to 3 and is a screening instrument rather than a diagnostic interview. The original validation reported high internal consistency (Kroenke et al., 2001). In this study, scores greater than 10 triggered exclusion. Because the conventional score band of 10–14 is often described as moderate symptom severity, retaining a score of exactly 10 should be distinguished from claiming that all moderate symptoms were excluded.

The purpose of PHQ-9 screening was to reduce, not eliminate, depressive-symptom heterogeneity. Mild or subclinical symptoms could remain in the formal sample, and anxiety and depression can co-occur. No causal attribution of music preference to trait anxiety alone is therefore warranted. Item-level PHQ-9 responses were not included in the inferential dataset used for this report, so a sample-specific internal-consistency coefficient was not recalculated.

The distinction among measures is therefore as follows: STAI-T and PHQ-9 are established multi-item psychological instruments; SAM-based valence and arousal ratings were used to annotate music stimuli; familiarity was a separate single study-specific rating; and the primary outcome was a separate 9-point preference rating collected after each music excerpt. These measures were not treated as interchangeable indicators of a single emotional construct.

#### 2.2.3 Experimental procedure

The formal experiment used a 3 (trait-anxiety group: high, medium, low) x 3 (valence-defined stimulus category: positive, neutral, negative) mixed design. Trait-anxiety group was a between-subjects factor and stimulus category was a within-subjects factor. Each participant rated all three categories, which reduced between-person baseline differences in rating scale use. The design does not isolate valence from arousal because the categories differed on both dimensions.

Operationally, high and low trait-anxiety groups were drawn from the upper and lower 27% of the eligible screening distribution, and the medium group from the central 46%. The formal experimental sample contained 30 participants per group. This grouping was used for the reported ANOVA and should not be interpreted as evidence that trait anxiety is naturally categorical. A continuous analysis would be preferable in a formal sample in which continuous STAI-T scores and trial-level preference outcomes are retained for every participant.

The procedure was implemented in PsychoPy, an open-source platform for constructing behavioral experiments with controlled stimulus presentation and response collection (Peirce et al., 2019), and delivered through the BrainIsland online platform. Participants provided electronic informed consent before beginning the experiment. They were asked to complete the session during a standardized morning window and were instructed to use headphones where possible and to set the computer audio to a moderate level. Because the study was conducted remotely, headphone use, device characteristics, room acoustics, and actual sound-pressure level could not be objectively verified.

Each session began with on-screen instructions and a 1-s fixation cross. The 90 excerpts were then presented in a participant-specific randomized order. After each 15-s excerpt, participants rated how much they liked the excerpt on a 9-point scale (1 = strongly disliked, 9 = strongly liked). A self-paced rest was available after every 30 clips. The session lasted approximately 20–25 min. Randomization and rest periods reduced systematic order effects, but trial position and within-session fatigue were not modeled statistically.

#### 2.2.4 Data analysis

Data were coded in Microsoft Excel and analyzed in SPSS 25.0. Descriptive statistics are reported as *M* ± SD, with 95% confidence intervals, skewness, and kurtosis additionally reported for the stimulus-level distributions in Table 1. For the primary analysis, the 30 preference ratings within each stimulus category were averaged for each participant, making the participant the unit of inferential analysis. A repeated-measures ANOVA was conducted with stimulus category as the within-subjects factor and trait-anxiety group as the between-subjects factor. Greenhouse–Geisser correction was used when sphericity was violated, and significant interactions were followed by simple-effects analyses with Bonferroni-adjusted comparisons. A second repeated-measures ANOVA examined gender as an exploratory between-subjects factor. This participant-level aggregation does not model random variation among individual music excerpts, and the reported model therefore generalizes most directly to the tested stimulus set. The analytic dataset available for the present study did not contain the complete participant-by-excerpt trial matrix needed for a crossed participant-and-stimulus mixed-effects model.

**Table 1:** Descriptive statistics of musical stimuli on three dimensions (*N* = 553 excerpts).

Dimension	Mean	SD	95% CI	Skewness	Kurtosis
Valence (Pleasantness)	4.79	1.00	[4.71, 4.87]	0.04	−0.58
Arousal	5.36	0.71	[5.30, 5.42]	−0.02	−0.48
Familiarity	4.36	0.81	[4.29, 4.43]	0.23	−0.39

CI = confidence interval. Values are reported at the stimulus level (*N* = 553). The 95% CIs are two-sided confidence intervals for the mean. Skewness values close to zero indicate approximate symmetry, whereas negative kurtosis values indicate relatively flatter, light-tailed distributions.

## 3 Experimental results

### 3.1 Results for the emotional music stimulus database

#### 3.1.1 Subjective ratings on three dimensions of musical stimuli

Subjective ratings were available for 553 validated excerpts, including 289 Chinese traditional/folk excerpts and 264 Western classical excerpts. Valence and arousal were SAM-based affective ratings; familiarity was a separate study-specific rating. Each dimension used a 9-point scale. Table 1 summarizes the stimulus-level descriptive statistics.

Mean valence was 4.79 (SD = 1.00, 95% CI [4.71, 4.87]), with near-zero skewness (0.04) and negative kurtosis (−0.58), indicating an approximately symmetric and relatively platykurtic distribution. Mean arousal was 5.36 (SD = 0.71, 95% CI [5.30, 5.42]), with skewness of −0.02 and kurtosis of −0.48. Mean familiarity was 4.36 (SD = 0.81, 95% CI [4.29, 4.43]), with mild positive skewness (0.23) and kurtosis of −0.39. Across the three dimensions, the small absolute skewness values and moderate negative kurtosis values indicate no marked distributional asymmetry at the stimulus level. Because valence and arousal were not orthogonalized in the stimulus pool, subsequent classification was descriptive rather than a factorial manipulation of two independent dimensions.

Familiarity varied across the full database and was higher for Chinese than Western excerpts, but familiarity did not differ across the three categories selected for the formal experiment. Thus, familiarity was checked as a potential confounding characteristic in the formal stimulus set, whereas arousal remained unequated.

Together, these statistics establish that the database spans a broad range of valence, arousal, and familiarity. They do not, however, demonstrate that valence can be interpreted independently of arousal. The formal preference findings are therefore reported at the level of valence-defined stimulus categories.

#### 3.1.2 Differences in valence, arousal, and familiarity between Chinese and Western music

Stimulus-level independent-samples *t*-tests compared Chinese traditional/folk and Western classical excerpts (Table 2). Chinese excerpts had slightly higher valence ratings (*M* = 4.89, *SD* = 0.99) than Western excerpts (*M* = 4.69, *SD* = 1.02), *t*(551) = 2.32, *p* = 0.02. Arousal did not differ significantly, *t*(551) = −1.49, *p* = 0.14. Chinese excerpts were rated as more familiar (*M* = 4.49, *SD* = 0.89) than Western excerpts (*M* = 4.22, *SD* = 0.68), *t*(551) = 4.06, *p* < 0.001. Cultural origin was therefore balanced within each formal category, and familiarity was checked across the selected categories.

**Table 2:** Stimulus-level differences in valence, arousal, and familiarity between Chinese and Western music.

Music type	Valence (*M* ± SD)	Arousal (*M* ± SD)	Familiarity (*M* ± SD)
Chinese Traditional	4.89 ± 0.99	5.32 ± 0.73	4.49 ± 0.89
Western Classical	4.69 ± 1.02	5.41 ± 0.68	4.22 ± 0.68
*t* (df = 551)	2.32	−1.49	4.06
*p*-value	0.02*	0.14	< 0.001***

*p ≤ 0.05, **p ≤ 0.01, ***p ≤ 0.001.

#### 3.1.3 Correlations among the three emotional dimensions of musical stimuli

Pearson correlations showed that valence was positively associated with arousal, *r* = 0.23, *p* < 0.01, and familiarity, *r* = 0.52, *p* < 0.01. Arousal was also positively associated with familiarity, *r* = 0.39, *p* < 0.01 (Table 3). These relationships indicate that the affective and familiarity characteristics of the stimuli were interrelated rather than statistically independent (Figure 3).

**Table 3:** Pearson correlation matrix for valence, arousal, and familiarity.

Measure	Valence	Arousal	Familiarity
Valence	1		
Arousal	0.23^**^	1	
Familiarity	0.52^**^	0.39^**^	1

*p ≤ 0.05, **p ≤ 0.01, ***p ≤ 0.001.

**Figure 3:**
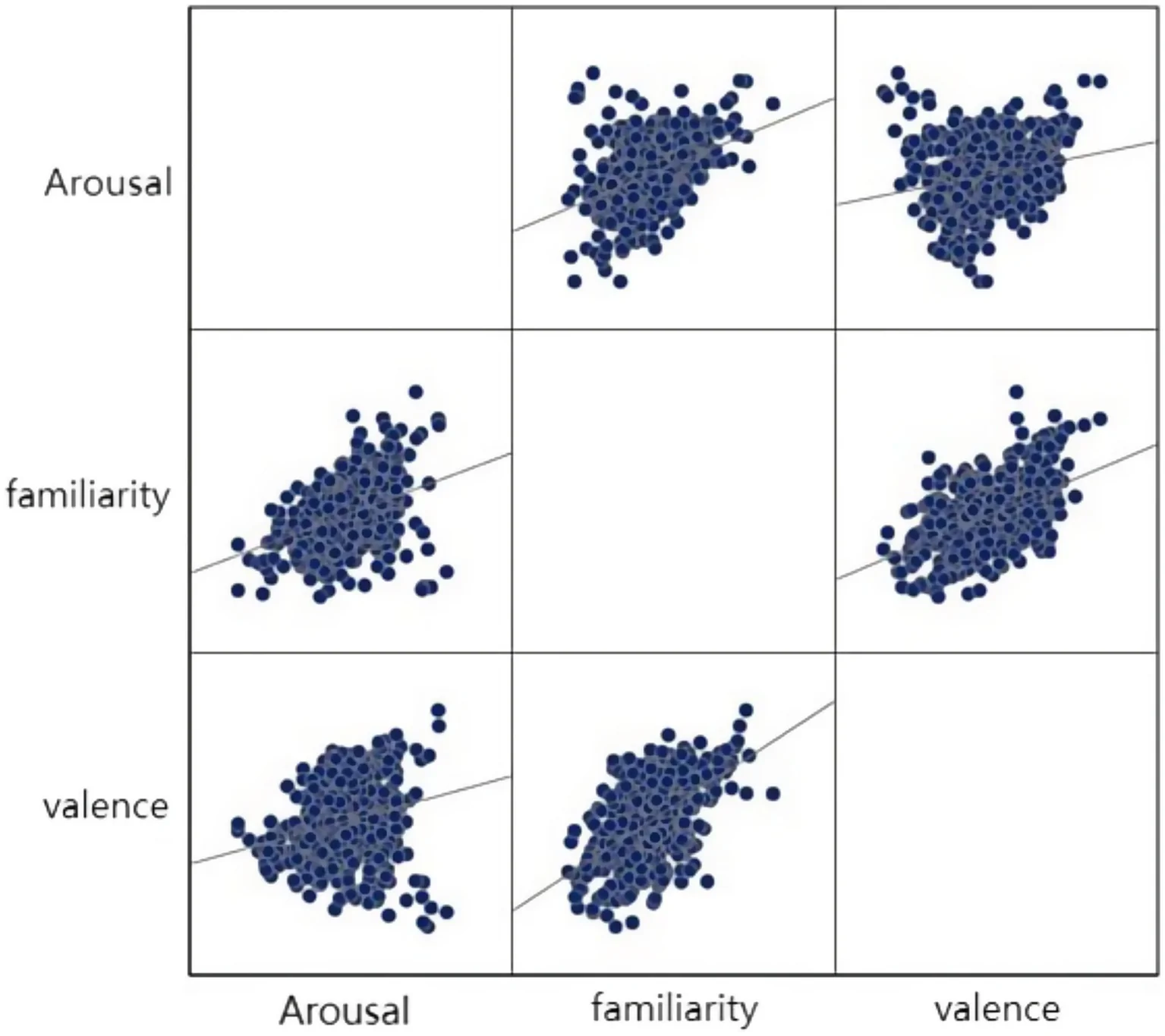
Stimulus-level relationships among valence, arousal, and familiarity.

### 3.2 Preferences across valence-defined music categories among college students with trait anxiety

#### 3.2.1 Descriptive statistics

Table 4 presents participant-level mean preference ratings. Across the formal sample, the positive category received the highest rating (*M* = 5.93, *SD* = 1.90), followed by the neutral category (*M* = 5.10, *SD* = 2.02) and the negative category (*M* = 3.77, *SD* = 2.08). The low trait-anxiety group had the highest mean for the positive category (*M* = 6.23, *SD* = 2.03), whereas the medium group had the highest mean for the negative category (*M* = 4.22, *SD* = 1.96). These are descriptive category differences; because arousal differed across categories, the means should not be interpreted as preference for valence independent of arousal.

**Table 4:** Participant-level descriptive statistics of preference ratings.

Trait anxiety group	Positive category (30 excerpts) (*n* = 30)	Neutral category (30 excerpts) (*n =* 30)	Negative category (30 excerpts) (*n =* 30)
	*M* ± *SD*	*M* ± *SD*	*M* ± *SD*
High Trait Anxiety	5.75 ± 1.81	5.02 ± 1.86	3.90 ± 1.96
Medium Trait Anxiety	5.82 ± 1.78	5.18 ± 1.88	4.22 ± 1.96
Low Trait Anxiety	6.23 ± 2.03	5.09 ± 2.02	3.20 ± 2.18
Total	5.93 ± 1.90	5.10 ± 2.02	3.77 ± 2.08

*n* = 30 participants in each trait-anxiety group; total *N* = 90. Each participant rated 30 excerpts in each stimulus category. Values are participant-level *M* ± SD.

#### 3.2.2 Main and interaction effects of trait anxiety group and stimulus category on preference

A two-way repeated-measures ANOVA tested trait-anxiety group (high, medium, low) as the between-subjects factor and valence-defined stimulus category (positive, neutral, negative) as the within-subjects factor. Participant-level category means were analyzed. Table 5 reports the main and interaction effects. The term stimulus category is used here because the conditions differed in arousal as well as valence.

**Table 5:** Repeated-measures ANOVA for trait-anxiety group x valence-defined stimulus category.

Effect	*F* (df)	*p*	Partial *η*^2^
Trait-anxiety group	2.48 (2,87)	0.090	0.054
Stimulus category	156.42 (1.96,170.60)	<0.001***	0.643
Group x stimulus category	7.13 (3.92,170.60)	<0.001***	0.141

*p ≤ 0.05, **p ≤ 0.01, ***p ≤ 0.001.

The main effect of trait-anxiety group was not significant, *F*(2, 87) = 2.48, *p* = 0.090, *partial η^2^* = 0.054. The main effect of stimulus category was significant, *F*(1.96, 170.60) = 156.42, *p* < 0.001, *partial η^2^* = 0.643. The trait-anxiety-group x stimulus-category interaction was also significant, *F*(3.92, 170.60) = 7.13, *p* < 0.001, *partial η^2^* = 0.141. Thus, the pattern of preference across the three tested categories varied among the predefined trait-anxiety groups.

Simple-effects analyses clarified the interaction. In the positive category, the group effect was significant, *F*(2, 87) = 3.11, *p* = 0.049, *partial η^2^* = 0.067; the low group rated the positive category higher than the high group after Bonferroni adjustment (*p* = 0.042). In the neutral category, the group effect was not significant, *F*(2, 87) = 0.06, *p* = 0.942, *partial η^2^* = 0.001. In the negative category, the group effect was significant, *F*(2, 87) = 3.42, *p* = 0.037, *partial η^2^* = 0.073; the medium group rated the negative category higher than the low group (*p* = 0.012), whereas the high-medium contrast was not significant after correction.

Within each trait-anxiety group, stimulus category had a significant effect: high group, *F*(1.94, 56.29) = 29.12, *p* < 0.001, *partial η^2^* = 0.501; medium group, *F*(1.91, 55.41) = 20.73, *p* < 0.001, *partial η^2^* = 0.417; low group, *F*(1.88, 54.52) = 55.36, *p* < 0.001, *partial η^2^* = 0.656. Positive-category ratings were highest in each group. The comparatively elevated negative-category mean in the medium group is a group-based pattern and cannot establish a nonlinear continuous relation between STAI-T and preference (Figure 4).

**Figure 4:**
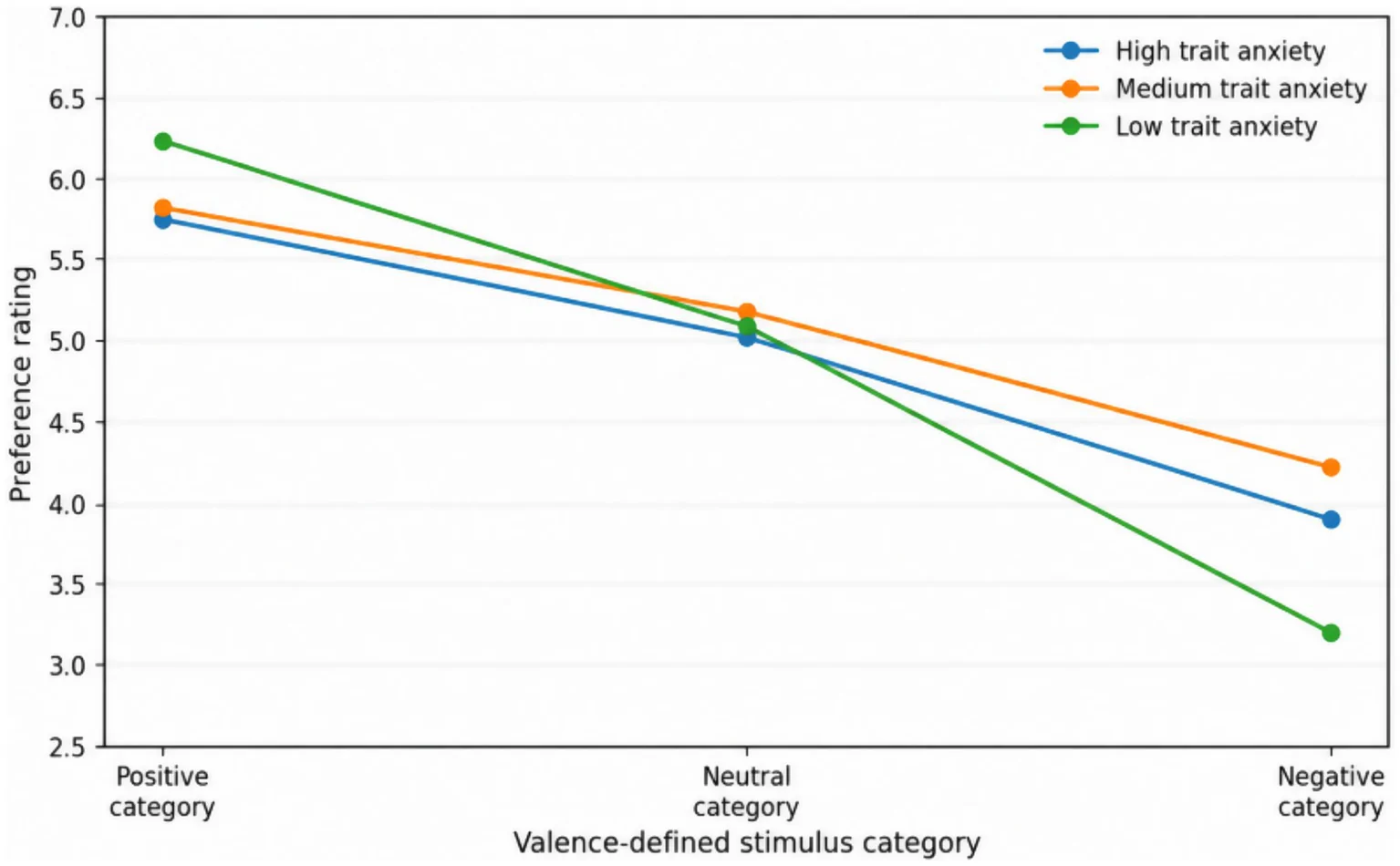
Interaction between trait-anxiety group and valence-defined stimulus category in preference ratings.

#### 3.2.3 Exploratory effects of gender on preference across stimulus categories

An exploratory repeated-measures ANOVA used gender (male, female) as the between-subjects factor and valence-defined stimulus category as the within-subjects factor. Participant-level category means were analyzed. Results are reported in Table 6.

**Table 6:** Repeated-measures ANOVA for gender x valence-defined stimulus category.

Effect	*F* (df)	*p*	Partial *η*^2^
Gender	4.24 (1,88)	0.043*	0.046
Stimulus category	95.41 (1.98,174.24)	<0.001***	0.520
Gender x stimulus category	0.47 (1.98,174.24)	0.626	0.005

*p ≤ 0.05, **p ≤ 0.01, ***p ≤ 0.001.

There was a small significant main effect of gender, *F*(1, 88) = 4.24, *p* = 0.043, *partial η^2^* = 0.046; males had a higher overall preference mean (*M* = 5.08) than females (*M* = 4.79). The stimulus-category effect was significant, *F*(1.98, 174.24) = 95.41, *p* < 0.001, *partial η^2^* = 0.520. The gender x stimulus-category interaction was not significant, *F*(1.98, 174.24) = 0.47, *p* = 0.626, *partial η^2^* = 0.005. Because gender was exploratory and the effect was small, no broad gender-specific mechanism is inferred.

##### 3.2.3.1 Hypothesis evaluation

H1 was supported at the category level: preference differed significantly across the three tested stimulus categories, with positive-category ratings higher than negative-category ratings. H2 was supported by the significant trait-anxiety-group x stimulus-category interaction. H3 was supported by the non-significant between-group effect in the neutral category alongside significant group effects in the positive and negative categories. These evaluations concern the tested valence-defined, arousal-differentiated categories; they do not establish effects of valence independent of arousal.

## 4 Discussion

### 4.1 Interpretation of the emotional music stimulus database

The database included Chinese traditional/folk and Western classical excerpts with subjective ratings of valence, arousal, and familiarity. Chinese excerpts were rated as slightly more pleasant and more familiar than Western excerpts, whereas arousal did not differ by cultural origin. Balancing cultural origin within each formal category and verifying similar familiarity across the selected categories reduced two sources of systematic bias. These controls should not be taken to mean that the categories differed only in valence.

Valence, arousal, and familiarity were positively correlated at the stimulus level, and the three formal categories differed markedly in arousal. This pattern is consistent with multidimensional accounts of musical affect (Russell, 1980; Juslin and Västfjäll, 2008) and with Ho and Loo’s (2023) emphasis on the interdependence of arousal, valence, tempo, familiarity, and preference. Hebb’s (1955) arousal formulation and Schachter and Singer’s (1962) two-factor account also caution against treating activation as irrelevant to affective evaluation. Consequently, the present results are more accurately described as preference differences across valence-defined, arousal-differentiated stimulus categories than as a pure valence-preference effect.

The database nonetheless provides a documented set of instrumental excerpts for studying music preference, particularly because lyrics were excluded and cultural origin was balanced in the formal set. Its principal methodological limitation is that tempo was not retained as a quantitative covariate and arousal was not matched across categories. Future stimulus construction should cross or match valence and arousal, record tempo in beats per minute, and use trial-level models that simultaneously include valence, arousal, familiarity, tempo, cultural origin, and other relevant acoustic descriptors.

### 4.2 Trait anxiety and preference across valence-arousal music categories

The central finding was an interaction between predefined trait-anxiety group and stimulus category. Low-trait-anxiety participants gave relatively higher ratings to the positive category, and the medium group rated the negative category higher than the low group. Neutral-category ratings did not differ among groups. These results establish a group-based association between trait anxiety and preference within the tested set; they do not identify which component of the category difference – valence, arousal, or their combination – produced the pattern.

The stronger positive-category preference in the low group is compatible with prior evidence that personality and dispositional characteristics covary with music preference (Rentfrow and Gosling, 2003; Vella and Mills, 2017). However, the present design did not measure an emotion-regulation goal or an emotional response produced by the music. The result should therefore be described as a preference pattern, not as evidence that low-anxiety listeners use positive music to regulate emotion.

The relatively higher negative-category rating in the medium group may be considered alongside self-verification and sad-music research (Swann et al., 1992; Arens and Stangier, 2020; Yoon et al., 2020; Wang et al., 2018), but such theories remain possible interpretations rather than measured mechanisms. In addition, the medium group was created from a restricted central STAI-T band and showed a narrow score distribution. The apparent medium-group pattern could therefore reflect a genuinely nonlinear association, the grouping procedure, or stimulus characteristics. A continuous trait-anxiety analysis in a larger formal sample is required before making claims about nonlinearity.

Gender was associated with a small difference in overall rating level, but the gender x stimulus-category interaction was not significant. Although prior work has reported gender-related differences in sad-music preference (Xu et al., 2021), the current result does not support a gender-specific category pattern. The gender finding is therefore treated as exploratory and should not be used to infer differences in emotional expression, coping style, or music-use motives that were not measured.

The theoretical concepts discussed in the Introduction – including self-verification and mood management – provide alternative reasons why listeners might select affect-congruent or affect-incongruent material (Swann et al., 1992; Zillmann, 1988). The present experiment did not directly measure these goals, catharsis, avoidance, mood repair, or state-anxiety change. Accordingly, the data cannot determine which of these processes, if any, explains the observed preference pattern.

The findings also do not establish therapeutic efficacy. Preferred music can be relevant to stress-related responses in other designs (Jiang et al., 2013, 2016), and music can be used for mood regulation in everyday life (Saarikallio and Erkkilä, 2007), but the current study did not administer a music intervention or measure symptom change. Its practical contribution is therefore limited to identifying preference patterns that may inform hypotheses for future intervention research rather than supporting personalized treatment recommendations.

For the same reason, neurobiological mechanisms are not inferred from these data. No physiological, neural, or autonomic measures were collected. Any future work on neural or physiological pathways should first establish robust behavioral associations using designs that independently characterize valence and arousal and that model both participant and stimulus variability.

Overall, the results suggest that trait-anxiety grouping is associated with how college students rated the tested music categories, while familiarity and cultural origin were partly controlled. The interpretation remains bounded by the multidimensional nature of the stimuli and by the use of grouped rather than continuous trait anxiety.

Accordingly, the main conclusion is deliberately narrow: among the 90 formal participants, preference patterns across the tested positive-, neutral-, and negative-valence categories differed by trait-anxiety group. Because the categories also differed in arousal and because emotion regulation was not directly measured, the study does not show that valence alone drives preference or that any category regulates or reduces anxiety.

### 4.3 Limitations and future directions

Several limitations constrain the interpretation and indicate priorities for future work.

First, trait anxiety was converted from a continuous STAI-T score into three groups, and only 30 participants from each band completed the formal preference task. The 373 eligible participants formed the screening pool; the 283 not selected for the formal experiment have no music-preference outcomes and therefore cannot be included in a preference model retrospectively. Grouping discards within-band information and can create or obscure non-linear patterns. Future studies should administer the full preference task to a larger sample and analyze STAI-T continuously, with nonlinear terms evaluated only when theoretically justified and adequately powered.

Second, the formal stimulus categories differed significantly in arousal as well as valence. Familiarity was similar across the final categories, cultural origin was balanced, and lyrics were excluded, but tempo was not quantified as a covariate and arousal was not matched. The category effect therefore cannot be attributed uniquely to valence. Future studies should use a factorial or matched stimulus design that crosses valence and arousal and should report tempo and other acoustic features explicitly.

Third, the primary analysis averaged 30 excerpts within each category for each participant. This approach treats participants as the inferential unit but does not model variability among individual excerpts. A more generalizable analysis would retain every trial and fit a mixed-effects model with crossed random effects for participant and stimulus, continuous STAI-T as a predictor, and fixed effects or covariates for valence, arousal, familiarity, tempo, cultural origin, gender, and trial position where available. The complete trial-level matrix required for such a model was not part of the inferential dataset used for this report, so no mixed-effects coefficients are presented.

Fourth, online administration limited control over headphones, playback hardware, sound level, and listening environment. Although presentation order was randomized and rests were provided, 90 trials may still have produced fatigue or rating drift, and trial position was not modeled. Fifth, sample-specific internal-consistency estimates for STAI-T and PHQ-9 could not be recalculated from the scale-total analytic dataset. Finally, the study did not measure state-anxiety change, mood change, emotion-regulation goals, catharsis, avoidance, treatment response, or neurobiological variables. Future research should retain item- and trial-level data, preregister the primary model, improve auditory standardization, and align conclusions strictly with directly measured outcomes.

## 5 Conclusion

In this sample of 90 Chinese college students, preference ratings differed across the three valence-defined, arousal-differentiated music categories, and the preference pattern varied across the predefined trait-anxiety groups. The low-trait-anxiety group showed relatively higher preference for the positive category, whereas the medium-trait-anxiety group rated the negative category higher than the low-trait-anxiety group; ratings for the neutral category did not differ significantly among groups. Because the stimulus categories also differed in arousal and the study measured preference rather than changes in mood or anxiety, these findings should be interpreted as category-specific associations rather than evidence of a causal emotion-regulation or therapeutic effect. Future studies using continuous STAI-T scores, valence-arousal-matched stimuli, and trial-level mixed-effects models can test the robustness and generalizability of these associations.

## Data Availability

The raw data supporting the conclusions of this article will be made available by the authors, without undue reservation.
